# Development and validation of the tool for the evaluation of the behavioral factors affecting the prevalence of musculoskeletal disorders in Iranian students

**DOI:** 10.1186/s12887-020-02452-8

**Published:** 2020-12-07

**Authors:** Mahin Nazari, Rokhsareh Beigi, Mahmood Salesi, Rosanna Cousins, Hamidreza Mokarami

**Affiliations:** 1 Department of Health Education and Promotion, School of Health, Shiraz University of Medical Sciences, Shiraz, Iran; 2 Student Research Committee, School of Health, Shiraz University of Medical Sciences, Shiraz, Iran; 3 Chemical Injuries Research Center, Systems biology and poisonings institute, Baqiyatallah University of Medical Sciences, Tehran, Iran; 4 Department of Psychology, Liverpool Hope University, Liverpool, UK; 5 Department of Ergonomics, School of Health, Shiraz University of Medical Sciences, PO Box 71645-111, Shiraz, Iran

**Keywords:** PRECEDE Model, Musculoskeletal Symptoms, Adolescence, Ergonomics, Behavioral Risk Factors, Health Promotion

## Abstract

**Background:**

This study was conducted with the aim of developing a standard and valid questionnaire to evaluate the behavioral factors affecting musculoskeletal disorders among adolescent students based on the educational and ecological diagnosis phase of the PRECEDE model.

**Methods:**

Based on the PRECEDE model and by using available resources and a panel of experts, a reservoir of items was proposed. The content validity of the questions was measured using content validity ratio (CVR) and content validity index (CVI). 400 Iranian first-year female high school students completed the questionnaire. The construct validity was assessed using confirmatory factor analysis (CFA). The reliability of the questionnaire was evaluated using Cronbach’s alpha coefficient.

**Results:**

The age range of study students was 13.69 ± 0.86 years. The final developed questionnaire included 25 items in three dimensions: knowledge (9 items), attitude (10 items) and enabling factors (6 items). The mean scores of CVI and CVR were 0.97 and 0.92, respectively. The results of CFA confirmed the three-factor structure of the questionnaire. The Cronbach’s alpha coefficients of the dimensions of knowledge, attitude and enabling factors were 0.65, 0.80 and 0.71, respectively.

**Conclusions:**

The present questionnaire had appropriate psychometric properties and could be used as a valid tool in evaluating the factors affecting the development of musculoskeletal disorders among adolescent students.

## Background

Adolescence is a critical period associated with many developmental changes. Adolescence is a time of transition, with many physical and psychological transformations needed in puberty to adapt and prepare boys and girls to enter adulthood. The set of changes made during this period and their influence on adolescent behaviors have an important impact on the formation of adolescent lifestyles [1, 2]. A World Health Organization study has provided robust evidence which indicates that combating high-risk behaviors and unhealthy habits in the teenage years has a significant positive impact on health in adulthood and aging [3]. Given the role of girls in fertility, it can be stated that care of the health of adolescent girls has a particular importance. The adolescence period for girls orients their later life and has a direct impact on their families and their children [4]. For this reason, the United Nations Population Fund has supported initiatives to improve girls’ health as a key factor in breaking the cycle of poverty among the generations [5].

Musculoskeletal disorders (MSDs) are nowadays recognized as an important health problem, with a relatively high prevalence in adolescents and students [6, 7]. MSDs are defined as health problems in the motor system, such as muscles, tendons, bones, cartilages, ligaments, and nerves [8–10]. The term includes minor transient complaints to irreversible damage and disability [11]. The prevalence of MSDs is between 16% and 86% in school children in developed countries, and in the range of 46.3–88.8% in developing countries [12].

The general factors affecting the prevalence of musculoskeletal pain in school children can be classified into three groups: (I) heavy school bags (weighing more than 10% of body weight), (II) disproportion between students’ anthropometric dimensions and school furniture, and (III) awkward sitting posture [7, 13]. Heavy school bags are harmful not only to the back and spinal cord, but also to other parts of the musculoskeletal system [6]. Sitting with poor body posture for a long time causes MSDs and high load mechanical stress [8]. It can also have a negative physiological effect, such as impaired blood circulation and reduced feelings of comfort, and it can cause psychological problems. These health issues can influence social interactions and relationship with peers, mental health, school absenteeism, academic competence, and participation in physical activity [14].

The literature also indicates that there are clear sex differences in presentation of MSDs. For example, a study of High School children in Scotland found girls suffered significantly more shoulder and neck pains and headaches than boys following extended periods of computer use [15]. A study in Iran found that although the weight of school backpacks and the average weight of boys and girls are almost the same up to age 13 years, where bag weight exceeded 10% body weight, MSDs were 1.44 times higher in girls than boys [16]. It has been proposed that girls are more susceptible to MSDs than boys due to the significantly earlier onset of puberty in females [17].

Given the current status of MSDs in students, there is a need for educational interventions to reduce harm to health in this regard. However, to obtain effective and useful results, any proposed educational intervention should be based on defined theories and models towards providing a systematic view of events and a systematic process for analyzing successes or failures [18, 19]. The PRECEDE model is one of the most effective and widely used models in various healthcare areas for health promotion [20, 21]. Its predictive power and reliability as a design tool and a framework for organizing and designing interventions in various social, behavioral, epidemiological and management sciences has been confirmed [22]. The PRECEDE model has been used in studies related to behavioral change in students to improve nutritional behaviors [23] and to promote safe traffic behaviors [24]. However, despite its potentials, to date this model has not been used to direct a MSDs prevention program. One important reason that is currently preventing its use is the lack of a standard and valid tool to evaluate the effective behavioral factors in musculoskeletal disorders in students based on this model. That is, it is necessary to evaluate the status of behavioral factors affecting musculoskeletal pain in students using valid and standard comprehensive tools and methods. These evaluations can then be used as a basis for designing and implementing targeted interventions and improving musculoskeletal pains in students. Hence, to address this fundamental gap, this study was carried out to design and develop a comprehensive standard tool for evaluating behavioral factors affecting MSDs in adolescent students based on the PRECEDE model.

## Methods

### Research design and population

A mixed methods design was used to develop a tool for evaluating the factors affecting the prevalence of MSDs among students. First, a qualitative study was conducted with the aim of identifying the factors related to MSDs in students using field observations, the views of a panel of experts, and a literature review. Then, a quantitative study was conducted to evaluate the psychometric properties of the tool being developed.

The study was conducted during the academic year 2018–2019 among all female first-year high school students in one of the Iran’s cities (Kavar). At the time of the study, a total of 590 students were studying in different schools of this city. Participation was voluntary and before distributing the questionnaire, the research objectives and its procedure were completely explained to all the students and their mothers. The students were ensured that their data would be analyzed only collectively. Written consent to participate was taken from 400 students and their mothers. Anonymous questionnaires were administered using face-to-face interviews. The research project was approved by the Scientific and Medical Ethics Committee of Shiraz University of Medical Sciences (IR.SUMS.REC.1398.007).

### Design of the questionnaire items

At the first stage, all the high schools of the city were studied by the research team and the risk factors related to MSDs were identified based on the PRECEDE model’s educational and ecological diagnosis phase. At this phase of the model, the factors influencing behavior were classified into three general groups of predisposing factors, enabling factors and reinforcing factors [25, 26]. The predisposing factors include “an individual’s knowledge, beliefs, values, and attitudes” [25]. The enabling factors include “programs, services, availability and accessibility of resources, or new skills required to enable behavior change” [25]. The reinforcing factors include the impact of others and their feedback (such as social support) [25]. In addition, the research literature was reviewed using various databases and the considered factors were extracted from related articles. Finally, an expert panel consisting of four ergonomic professors, two occupational health professors, and four health education professors was formed. Panel members discussed influential factors, developed a list, and classified and prioritized the causative factors for each behavioral goal. They reached a consensus for each of three dimensions of ​​the questionnaire, with respect to the identified risk factors. Then, appropriate initial items were formulated.

The initial version of the questionnaire consisted of 34 items in three dimensions: knowledge (14 items), attitude (13 items), and enabling factors (7 items). The knowledge dimension included items on students’ knowledge of the proper weight, and arrangement of backpack items, and the characteristics of the shoulder strap; and the proper posture for studying and doing the assignments. The attitude dimension included items on students’ attitude about weight, type and way of carrying the school bag, status of school bag straps and cushion, and the effect of posture on body organs during study and doing assignments on the prevalence of the MSDs. Enabling factors included items on having access to resources such as training sessions on MSDs and ergonomic principles in purchasing and carrying bags and on the proper posture of one’s body during studying and doing assignments. To increase face validity, it was determined that items have these properties: (1) shortness, (2) clarity, (3) no negative verbs and, if needed, the items itself should be negative, (4) single-part, and (5) no leading questions.

### Examining the psychometric properties of the tool

To ensure the results of measurement using the new student’s MSD tool would be useful and accurate, the questionnaire was evaluated in terms of validity and reliability.

### Face and content validity

The ten professors that made up the experts panel were asked to examine the grammar, wording, and item allocation of the student’s MSD tool. If the given principles were not observed, they would be asked to suggest a correction for the items. In addition, in order to eliminate any possibility of ambiguity and promote easy understanding of the items, the views of 20 students were obtained and their considered corrections were applied to the items.

Content validity index (CVI) and content validity ratio (CVR) were used to evaluate the content validity. To examine the CVI, the ten experts were asked to examine the three criteria of relevancy, clarity, and simplicity separately for each item [27]. Based on the guidelines, a CVI more than 0.79 was considered appropriate, between 0.7 and 0.79 needed to be reviewed and less than 7 was considered unacceptable and had to be eliminated [27]. The Lawshe method was used to examine CVR [28]. To study this index, the panel of experts were asked to examine the necessity of each item. Based on the Lawshe’s table, which accounts for the number of experts on a panel, items with a CVR of more than 0.62 (for 10 experts) were necessary for inclusion; items with a lower CVR were eliminated [28].

### Construct Validity

Confirmatory factor analysis (CFA) with the Likelihood Maximum method was used to examine construct validity according to the PRECEDE model’s educational and ecological diagnosis phase and identified questionnaire dimensions. CFA is a statistical technique that tests hypothesized models. Simultaneous analyses of all variables in a model are examined to explore whether the model is consistent with the data. CFA allows models to be driven both statistically and theoretically, which traditional multivariate procedures like exploratory factor analysis (EFA) are unable to do [29]. The sample size was sufficient for factor analysis as it fell into the necessary 4 to 10 times more than the number of variables [30]. Chi-Square/Degrees of Freedom Ratio (χ2/*df*), Root Mean Square Error of Approximation (RMSEA), Goodness-of-Fit Index (GFI) and Adjusted Goodness-of-Fit Index (AGFI), and Comparative Fit Index (CFI) were used to measure goodness-of-fit of the CFA model [30, 31]. Values of the χ2/*df* ratio 2 or a smaller were considered a good fit [32, 33]. Values of RMSEA smaller than 0.08 were considered an acceptable fit and lower than 0.05 were considered a good fit [30, 32]. Values of GFI and AGFI greater than 0.8 or 0.9 were considered as a good fit [30, 34]. Finally, values of CFI greater than 0.9 were considered as a good fit [34].

### Reliability

Reliability was estimated through calculation of internal consistency (Cronbach’s alpha coefficient). An alpha value between 0.6 and 0.7 has been considered as acceptable level of reliability [35]. Taber also introduced alpha value 0.58–0.97 as the satisfactory level of reliability in a review study of educational instruments [36]. Following Taber, in this study, an alpha value above 0.6 was considered as the acceptable level.

The collected data were analyzed using IBM SPSS 23 and AMOS 23 (USA, SPSS Inc.)

## Results

The study population comprised 400 female students aged 13.96 ± 0.86 years, height 161.08 ± 5.99 cm, weight 52.89 ± 9.62 kg and a body mass index of 20.35 ± 3.31 kg/m^2^.

### Tool validity

The changes suggested by the panel of experts and the students were readily applied to the items of questionnaire to support face and qualitative content validity. Based on the results of CVI and CVR, 2 items (1 ‘attitude’ and 1 ‘knowledge’) were eliminated. The mean score of total CVI value of the remaining 32 items was 0.96 and the mean CVR value was 0.92.

Before performing factor analysis, the correlation coefficient between each item score and the total score in each dimension was examined. The results showed that corrected item total correlation of two items of attitude dimension (‘in my opinion, implementation of ergonomic principles of carrying bags prevents musculoskeletal disorders’, *r* = 0.10; and, ‘in my opinion, performing appropriate exercises prevents musculoskeletal disorders’, *r* = 0.17), four items of knowledge dimension (‘in your opinion, what is the proper weight for a backpack?’, *r* = 0.02; ‘what are the features of an appropriate backpack shoulder straps?’, *r* = 0.11; ‘what is the best waist posture when doing assignments?’; *r* = 0.13; and ‘what is the maximum time of continual sitting when doing assignments?’, *r* = 0.18), and one item of the enabling dimension (‘is it possible for you to purchase an appropriate backpack?’, *r* = 0.07) did not have the discrimination power required for measuring the considered dimensions. Therefore, these items were eliminated before implementation of the CFA.

**Fig. 1:**
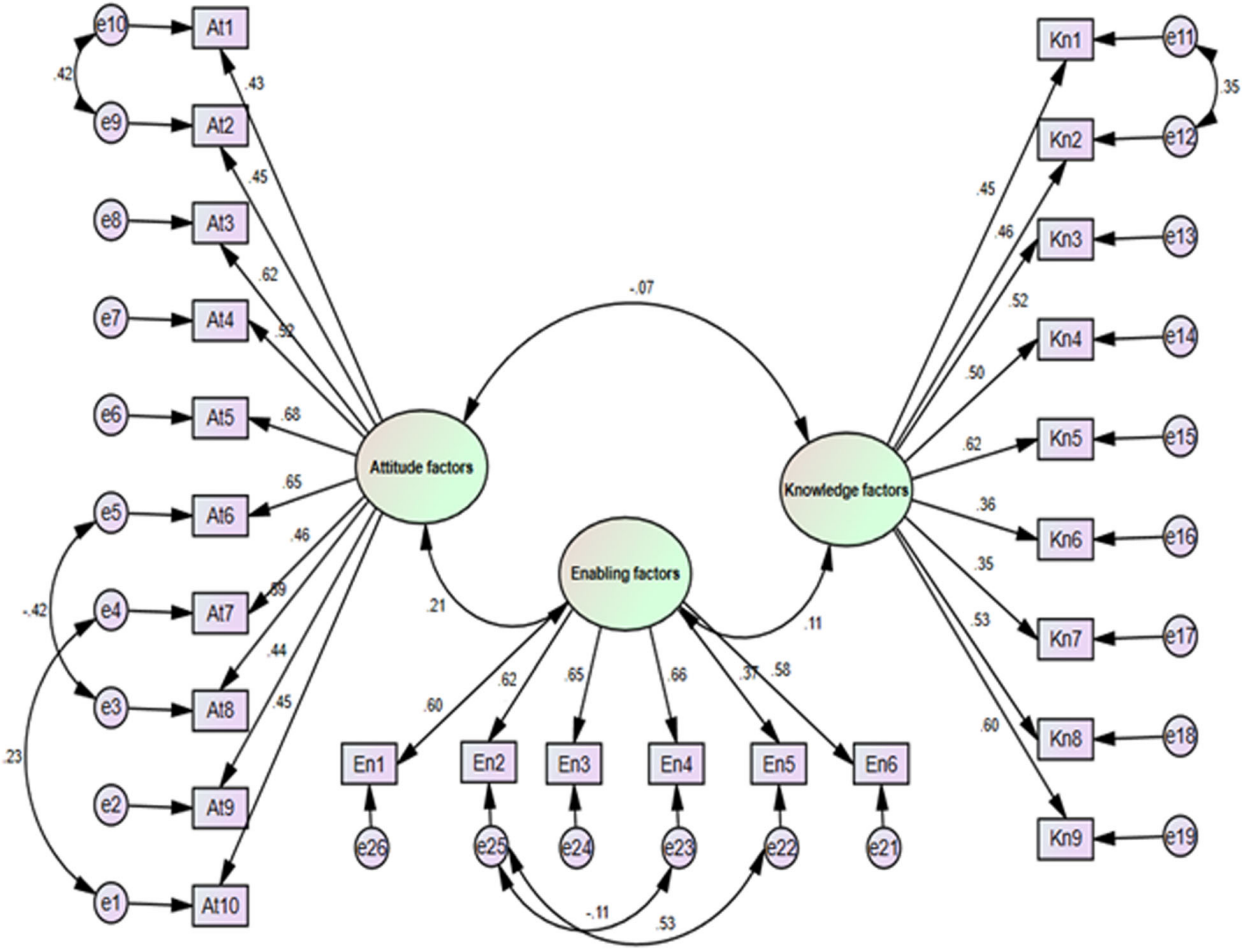
Final modified model

The results of the CFA showed that the fit indices of the default model were not suitable. Therefore, in order to achieve a satisfactory fit of the model with the data, the model was improved by releasing some parameters based on the proposed adjustment modification indices through the AMOS software. Figure 1 illustrates the path diagram of the CFA after releasing these parameters along with the standardized factor loadings of the items. All factor loadings of the items were significant in all three dimensions. The goodness-of-fit indices were as follows: χ2 = 493 (*df* = 266), χ2/*df* = 1.85; GFI = 0.90; AGFI = 0.88; CFI = 0.90; and RMSEA = 0.049.

According to fit indices and standardized coefficients and the Critical Rate Index (CRI((Table 1), the final model had an acceptable fit. Measurement error of items At1 with At2, At6 with At8, and At7 with At10 of Attitude dimension, measurement error of items En2 with En4, and En2 with En5 of Enabling Factors dimension, and measurement error of items Kn1 and Kn2 of Knowledge dimension were correlated. Moreover, these items had the highest Cronbach’s alpha if item deleted and the least corrected item-total correlation compared to other items.

**Table 1:** Items loading factor and critical rates of dimensions of questionnaire

Dimension	Item	Standardized Regression Weight	Critical Rate	*p*
Attitude	At1	0.431	5.94	< 0.001
	At2	0.455	6.14	< 0.001
	At3	0.617	7.24	< 0.001
	At4	0.523	6.67	< 0.001
	At5	0.679	7.54	< 0.001
	At6	0.648	7.23	< 0.001
	At7	0.461	7.08	< 0.001
	At8	0.594	6.93	< 0.001
	At9	0.435	5.99	< 0.001
	At10	0.453	-	-
Knowledge	Kn1	0.453	-	-
	Kn2	0.461	3.36	< 0.001
	Kn3	0.519	3.579	< 0.001
	Kn4	0.497	3.551	< 0.001
	Kn5	0.618	3.039	< 0.001
	Kn6	0.361	3.249	0.001
	Kn7	0.350	3.213	0.001
	Kn8	0.535	3.599	< 0.001
	Kn9	0.604	3.020	< 0.001
Enabling factors	En1	0.598	7.73	< 0.001
	En2	0.623	3.54	< 0.001
	En3	0.647	8.04	< 0.001
	En4	0.660	8.09	< 0.001
	En5	0.369	5.44	< 0.001
	En6	0.578	-	< 0.001

### Reliability

The Cronbach’s alpha coefficients showed that the reliability of all three dimensions was appropriate. The relationship of items with the total score of each area was also appropriate. Cronbach’s alpha of each dimension and mean score, corrected item-total correlation, and Cronbach’s alpha if item deleted of items of each dimensions are presented in Table 2.

**Table 2:** Reliability of the dimensions of questionnaire

Dimension	Item	Corrected Item-Total Correlation	Cronbach’s Alpha if Item Deleted	Cronbach’s Alpha (CI95%)
Attitude	At1	0.46	0.78	0.80 (0.76–0.83)
	At2	0.49	0.78	
	At3	0.60	0.76	
	At4	0.45	0.78	
	At5	0.58	0.77	
	At6	0.50	0.78	
	At7	0.39	0.79	
	At8	0.47	0.78	
	At9	0.36	0.79	
	At10	0.42	0.78	
Knowledge	Kn1	0.35	0.59	0.61 (0.54–0.67)
	Kn2	0.31	0.59	
	Kn3	0.45	0.56	
	Kn4	0.44	0.56	
	Kn5	0.32	0.59	
	Kn6	0.34	0.58	
	Kn7	0.33	0.59	
	Kn8	0.45	0.55	
	Kn9	0.30	0.60	
Enabling	En1	0.42	0.65	0.71 (0.63–0.73)
	En2	0.38	0.67	
	En3	0.40	0.66	
	En4	0.47	0.62	
	En5	0.51	0.61	
	En6	0.41	0.64	

The Cronbach’s alpha of Attitude, Knowledge and Enabling Factors were 0.80 (CI95%: 0.76–0.83), 0.61(CI95%: 0.54–0.67) and 0.71 (CI95%: 0.63–0.73), respectively. These values suggested an acceptable level. None of the Cronbach’s alpha if item deleted of items was greater than the Cronbach’s alpha values of the each of the three dimensions. This meant that none of the items needed to be removed.

The final Tool for the evaluation of behavioral factors affecting musculoskeletal disorders in students is presented in Appendix 1.

## Discussion

In this study, for the first time, a tool was developed to evaluate behavioral factors affecting the MSDs in adolescent students based on the PRECEDE model’s educational and ecological diagnosis phase. The final questionnaire included 25 items and three dimensions: knowledge (9 items), attitude (10 items) and enabling factors (6 items). The results of the analyses of face validity, content validity, construct validity and reliability indicated the appropriateness of the psychometric properties of the designed questionnaire.

This tool was developed because the prevalence of MSDs in adolescents is unnecessarily high [37], and a means of undertaking evidence-based interventions to reduce this health risk is needed. Growing children are not sufficiently developed in terms of their skeletal and bone structure to tolerate the stress and physical demand of carrying a bag around school that weighs more than they do [38] or of extended periods of being hunched over a computer [15] at the expense of sufficient exercise [37]. Interventions to remove the predisposing factors of MSDs and prevent the pains associated with these behaviors are called for to improve the health and wellbeing of students. To achieve this goal, a standard tool is vital. The tool designed in this study could be used towards meeting this need.

To develop the tool, the views of ergonomics, occupational health and health education experts and the views of students were used to assess the face validity and qualitative content validity of the questionnaire, and the considered changes were applied to increase the validity of the questionnaire. In addition, CVI and CVR indices were used to evaluate the content validity. The obtained results indicated the validity of the questionnaire in terms of these two indices [30]. Moreover, the results of the CFA indicated that the factor structure of the three dimensions defined for the questionnaire was acceptable. This method of analysis tests the optimal match and fit of the observed and theoretical factor structures for the data set after identifying the pre-empirical factors (based on using PRECEDE model), by evaluating the fit of the pre-defined factor model. The fit indices of the evaluated model in this study included χ2/*df*, RMSE, GFI, AGFI, and CFI; these indices indicated a good fit of the three dimensions of the questionnaire [30–33]. Additionally, calculated factor load (standardized regression weight) of the items of all three dimensions of the questionnaire was significant (based on the CRI), indicating the appropriateness of the designed to measure the dimensions [39].

Reliability indicates consistency of a measure and its stability. It indicates how much the questionnaire can be repeated, or if repeatedly used the extent to which the same unit of analysis will bring the same results [40]. The results of Cronbach’s alpha coefficient, as the most widely used methods for measuring reliability, indicated that the internal consistency of all three areas of the questionnaire was appropriate. There was also a good correlation between the score of each item and the total score of each dimension. Both of these indices suggested the good reliability of the designed questionnaire [36].

It should be noted that reinforcing factors were not investigated in this study due to the individual nature of the behavior of carrying a backpack and sitting at a computer, and at this stage we do not know the influence of these factors on the prevalence of MSDs in adolescents. Nevertheless, this new tool provides a means of driving an intervention agenda to reduce the rates of MSDs in adolescents. There are various studies documenting the musculoskeletal injury risks of children and adolescents in the way they perform necessary activities, and they generally conclude that their findings provide a focus for intervention. The tool developed in this study could supportive of initiatives to reduce the prevalence of MSDs in growing children.

### Limitations of study

We acknowledge that this research has some limitations. First, this study was conducted only on a population of female adolescent students. This decision was made as in the first instance, the rates of MSDs are significantly higher in girls than boys at this time of life. Second, due to the wide scope of the subject, all components of the PRECEDE model were not studied. PRECEDE’s educational and ecological diagnosis phase was selected on the basis that it was most relevant according to the research literature and views of the experts. It remains, however, that they may be other aspects of the model that need to be considered to manage rates of adolescent MSDs. Third, although the factor load of items of the three dimensions of questionnaires was significant and acceptable, some items did not have a high load factor coefficient. It remains, however, that the items were psychometrically robust, and were derived though robust methodological procedures. Fourth, due to financial and executive limitations, this study was carried out only in one city in Iran. We endorse further studies using the tool to confirm its efficacy.

## Conclusions

The results of this study led to the development of a comprehensive and standard questionnaire for evaluating the factors affecting the prevalence of MSDs in students based on the PRECEDE model’s educational and ecological diagnosis phase. The proposed questionnaire had appropriate psychometric properties and could be used as a valid and practicable tool for evaluating the factors affecting the prevalence of MSDs and the implementation of targeted interventions among the students. This questionnaire can be used as a self-administered tool to assess behavioral factors affecting musculoskeletal disorders among adolescence students. Researchers can also use this tool to support and evaluate the effect of intervention programs to reduce adolescent MSDs.

## Data Availability

The datasets used and/or analyzed during the current study are available from the corresponding author on reasonable request.

## Appendix

**Table 3:** The final Tool for the Evaluation of Behavioral Factors affecting Musculoskeletal Disorders in Students.

**Dimensions**	**Items**
**Attitude**	1. In my opinion, the weight of the backpack is not so important as to cause musculoskeletal problems.
	2. In my opinion, the type of school bag is not so important as to cause musculoskeletal problems.
	3. In my opinion, improper carrying of a school bag is not so important as to cause musculoskeletal problems.
	4. In my opinion, backpack shoulder strap belt cushions pads do not have any effect on preventing musculoskeletal problems.
	5. In my opinion, adjusting the backpack straps is not so important as to cause musculoskeletal problems.
	6. In my opinion, the use of backpack chest and waist straps is not important enough to prevent musculoskeletal problems.
	7. In my opinion, head and neck posture during studying and doing homework has no effect on musculoskeletal problems.
	8. In my opinion, back posture during studying and doing homework has no effect on musculoskeletal problems.
	9. In my opinion, legs posture during studying and doing homework has no effect on musculoskeletal problems.
	10. In my opinion, the duration of sitting activities has no effect on the development of musculoskeletal problems.
**Knowledge**	1. What is the best way to carry backpack?
	2. Which type of bag is best for back and spine health?
	3. What should be the length of a backpack
	4. How should the backpack be organized?
	5. What kind of straps should a standard backpack have?
	6. What is the safest way to lift a backpack?
	7. What do you do if your school supplies are heavy?
	8. What is the proper sitting (back) posture during studying or doing homework?
	9. What is the proper way to position feet during studying or doing homework on a bench or chair?
**Enabling**	1. Have you been trained in preventing musculoskeletal problems?
	2. Have you been reminded because of inappropriate way if carrying your backpack (by family, friends, or teachers)?
	3. Have you been trained in the ergonomics principles for buying a backpack?
	4. Have you been trained in a proper sitting posture for studying or doing homework?
	5. Have you been reminded if you sitting in an awkward posture?
	6. Have you been trained in proper exercise to prevent musculoskeletal problems?

## Abbreviations

MSDs: Musculoskeletal disorders
CVR: Content validity ratio
CVI: Content validity index
CFA: Confirmatory factor analysis
χ2/df: Chi-Square/Degrees of Freedom Ratio
RMSEA: Root Mean Square Error of Approximation
GFI: Goodness-of-Fit Index
AGFI: Adjusted Goodness-of-Fit Index
CFI: Comparative Fit Index
